# Efficacy of Lasers in Debonding Ceramic Brackets: Exploring the Rationale and Methods

**DOI:** 10.7759/cureus.61050

**Published:** 2024-05-25

**Authors:** Ahmed S Khalil, Fisal A Baowideen, Ashraf S Alhujaili, Nasser F Alotaibi, Waleed A Almanjhi, Hanouf Y Yassin, Mohammad M Nasser, Ahmed F Alzahrani, Rawan S Alrehaili

**Affiliations:** 1 Orthodontics, Private Practice, Alexandria, EGY; 2 Dentistry, King Abdulaziz University, Jeddah, SAU; 3 Dentistry, Ministry of Health, Medina, SAU; 4 Dentistry, King Fahad Military Medical Complex, Dhahran, SAU; 5 Dentistry, King Khalid University, Abha, SAU; 6 Dentistry, Batterjee Medical College, Jeddah, SAU; 7 Dentistry, Your Health Medical Center, Taif, SAU; 8 Dentistry, Private Practice, Medina, SAU

**Keywords:** laser-aided debonding, debonding, ceramic bracket, bracket, laser

## Abstract

The development of ceramic brackets in orthodontics three decades ago emerged as a response to the increasing patient demand for less visible orthodontic appliances. While these brackets provide superior aesthetics, they are characterized by lower fracture toughness and higher bond strength in contrast to metal brackets. These properties present challenges during the debonding step, including the risk of enamel micro-fractures and cracks. Historically, various strategies have been developed to address challenges associated with debonding, reduce patient discomfort, and ensure that the bond failure site is confined to the bracket-adhesive interface. This included the use of specially designed debonding pliers, electrothermal debonding, ultrasonic technique, and chemical agents. Recently, there has been a shift towards utilizing different types of laser irradiation for this purpose. The burgeoning strategy, however, requires diligent scientific scrutiny to establish a standardized protocol with particular laser parameters and ultimately achieve the goal of enhancing the patient experience by reducing discomfort. This article offers a narrative review of laser-aided debonding of ceramic brackets, aimed at comparing different laser types, presenting their benefits and downsides, validating the efficiency of each method, and summarizing the published literature on this subject. It also provides insights for orthodontists on reducing patient discomfort that usually accompanies debonding ceramic brackets by delving into the science behind the use of lasers for this purpose

## Introduction and background

The invention of ceramic brackets in orthodontics approximately 30 years ago was a consequence of the growing demand among patients for less conspicuous orthodontic appliances [1]. While they offer enhanced esthetics, ceramic brackets demonstrate decreased fracture toughness and increased bond strength relative to metal brackets [2]. These characteristics pose challenges during debonding such as enamel micro-fractures and cracks [3]. Across the annals of history, diverse endeavors were suggested to reduce patient discomfort encountered, address the concerns of enamel damage, and opt for bond failure restricted primarily to the bracket-adhesive interface. Initially, specially manufactured pliers were proposed for the mechanical debonding of ceramic brackets [4]. Yet, the immoderate force along with patient discomfort remained inevitable [5,6].

The concept of using regulated heat application was suggested as an electrothermal approach for debonding ceramic brackets via thermal softening of adhesive [7]. However, it gave rise to the concern about potential pulpal injury [4]. Later, the debonding of ceramic brackets with the aid of ultrasonic tips showed promising results with the advantage of utilizing them later for the removal of the remaining adhesive [8]. Nevertheless, one of the drawbacks continued to be the notable increased debonding time [9]. Recently, the debonding of ceramic brackets with the use of laser application was assessed in diverse research. Examples included carbon dioxide (CO_2_) laser [10-13], neodymium-doped yttrium-aluminium garnet (Nd:YAG) [14,15], diode laser [16-19], erbium, chromium-doped yttrium, scandium, gallium and garnet (Er,Cr:YSGG) [20-22], and erbium-doped yttrium aluminum garnet (Er:YAG) [23-26].

The idea of utilizing debonding using lasers was primarily elucidated as thermal ablation or photoablation [25]. Others explained it as the thermal softening of adhesive. Laser output, wavelength, mode, lasing time, and type of adhesive varied across the studies. In essence, Er:YAG and Er,Cr:YSGG lasers demonstrated superiority compared to Nd:YAG and CO_2_ lasers, with the capability to be absorbed by the adhesive without posing harm to pulpal tissues [27]. One benefit of the diode laser was its compact size and lightweight. Nevertheless, conflicting results were reported with regard to the effectiveness of diode lasers in debonding ceramic brackets [16,17,28]. The lack of a defined methodology with laser parameters further complicated it [19,29]. 

**Figure 1:**
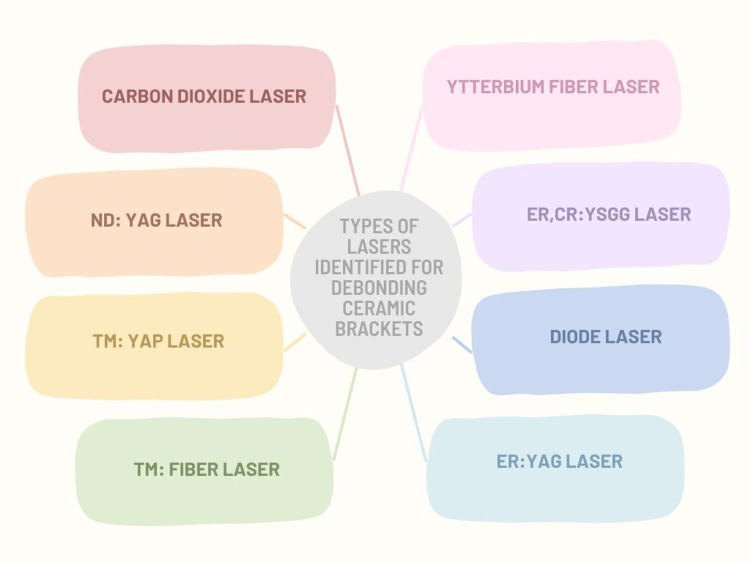
Types of lasers identified for debonding ceramic brackets ND:YAG: neodymium-doped yttrium-aluminium garnet; TM:YAP: thulium-doped yttrium aluminum perovskite; ER,CR:YSGG: erbium, chromium-doped yttrium, scandium, gallium and garnet; ER:YAG: erbium-doped yttrium aluminum garnet; TM: thulium Image credit: Ahmed S. Khalil

This article offers a narrative review of laser-aided debonding of ceramic brackets, identifies the various lasers used for this purpose (Figure 1), and comprehensively summarizes the published literature on this subject.

## Review

CO_2_ laser

Strobl et al. conducted a study to compare the debonding of monocrystalline and polycrystalline ceramic brackets in terms of bracket breakage, residual adhesive, and enamel damage [30]. They used two different adhesive pastes to bond ceramic brackets onto a sample of 93 extracted incisors and molars. In the experimental group, a CO_2_ laser was applied for two seconds with an additional torquing force averaging 22.8 N. The authors described the debonding mechanism as thermal softening. Their findings indicated that using a CO_2_ laser significantly reduced the force needed for debonding, consequently lowering the risk of enamel cracks compared to traditional debonding methods using pliers.

In the same vein, Mimura et al. examined the debonding characteristics of ceramic brackets using two different adhesives: bisphenol A-glycidyl methacrylate (Bis-GMA) and methyl methacrylate (MMA) resin [31]. The study involved 123 extracted premolar teeth. Teeth were cleaned, etched, rinsed, and dried before bonding polycrystalline ceramic brackets according to the adhesive manufacturers' instructions. The application of CO_2 _laser at 3 and 7 watt (W) was tested, and both shear bond strength (SBS) and adhesive remnant index (ARI) were measured. Results indicated a higher likelihood of adhesive remnants on enamel with MMA, suggesting that MMA provided a safer debonding process with lasers compared to Bis-GMA.

Ma et al. compiled a study to develop a technique aimed at reducing the breakage of ceramic brackets during the debonding process [32]. Their study involved 30 extracted bovine deciduous mandibular incisor teeth, which were prepared by pumicing, etching, rinsing, and drying. Polycrystalline ceramic brackets were then bonded to the labial surfaces of the teeth using a conventional adhesive system as per the manufacturer's instructions. A CO_2_ laser with a 20 W output was utilized in conjunction with debonding pliers for the debonding process. The laser tip was positioned perpendicularly and as close as possible to the bracket surface. The findings indicated that debonding pliers were safe to use, and the application of the CO_2_ laser effectively reduced the tensile strength by thermally softening the adhesive.

Obata et al. conducted a study to evaluate the debonding efficacy of CO_2_ lasers in super pulse and normal pulse modes for polycrystalline ceramic brackets [11]. The experiment utilized 230 extracted human premolar teeth that were cleaned, polished, rinsed, and dried. After bonding ceramic brackets and storing them for 24 hours at 37°C, the experimental group's brackets were irradiated with a CO_2_ laser at a wavelength of 10.6 μm. Subsequent shear tests conducted with a universal testing machine revealed that the super pulse mode was more effective for debonding. This caused fewer adverse effects on pulpal tissues.

Iijima et al. explored the effects of CO_2_ laser debonding on the mechanical properties of enamel, such as elasticity and hardness [12]. They used 53 extracted human premolars, which were prepared, bonded with monocrystalline ceramic brackets using conventional adhesives, and then stored in artificial saliva at 37°C for 24 hours. The study divided the teeth into groups using conventional and self-etching adhesives, applying various laser outputs (3, 4, 5, and 6 W). The CO_2_ laser, emitting a continuous wavelength of 10.6 μm, was applied for five seconds. The results indicated that higher laser outputs resulted in lower SBS, with the most significant reduction observed at the highest output settings

In another investigation, Tehranchi et al. assessed the performance of a CO_2_ laser in the debonding of polycrystalline ceramic brackets compared to conventional methods [33]. Thirty extracted human premolar teeth were bonded with a chemically cured composite, followed by the application of a CO_^2^_ laser in super pulse mode at 50 W for five seconds. The laser was positioned at a calculated distance from the center of each bracket. Shear testing was performed at a rate of 1 mm/minute, and the ARI was evaluated. The findings showed a significant reduction in SBS in the lased group, leading to less enamel damage as the debonding primarily occurred at the bracket-adhesive interface. The authors concluded that the CO_2_ laser's high absorbability by ceramic materials makes it an optimal choice for debonding. This could minimize potential damage to enamel and surrounding tissues.

Ahrari et al. conducted a comparative study examining the direction, length, and frequency of enamel cracks during the debonding process across four different groups [34]. These groups included mechanically retentive monocrystalline ceramic brackets debonded with a CO_2_ laser, mechanically retentive monocrystalline ceramic brackets debonded using conventional pliers, chemically retentive polycrystalline ceramic brackets debonded with a CO_2_ laser, and chemically retentive polycrystalline ceramic brackets debonded using conventional pliers. The study utilized 90 extracted human premolar teeth, which were prepared and then bonded with brackets using a conventional adhesive system. For the laser-assisted groups, a CO_2_ laser with a wavelength of 10.6 μm and ultra pulse mode was applied at a distance of 5 mm for five seconds, using a scanning method. The results indicated an increased incidence of bracket fracture and enamel cracks in the groups debonded with conventional pliers. This prompted the authors to suggest laser debonding as a method to minimize enamel damage.

Saito et al. explored the effectiveness of laser-assisted debonding of ceramic brackets using an experimental adhesive containing microcapsules with thermal expansion properties [35]. The study involved 96 extracted bovine mandibular incisor teeth, which were prepared, bonded with ceramic brackets and stored in distilled water at 37°C for complete polymerization. In the experimental setup, a CO_2_ laser with an output of 3 W was applied directly to the bracket's labial surface. Shear tests were conducted along with an assessment of ARI. The study found that SBS decreased significantly when the CO_2_ laser was applied for longer durations, with minimal differences in adhesive remnants. The findings suggested that combining CO_2_ laser with thermally expansive microcapsule adhesive could yield beneficial outcomes with reduced enamel damage.

Lastly, Macri et al. undertook a study to evaluate the SBS and residual adhesive on polycrystalline brackets [13]. Seventy-five extracted premolar teeth were prepared, bonded using a self-etching adhesive system, and stored at 37°C for 24 hours. Various CO_2_ laser settings were tested, including different outputs (5, 8, and 10 W), pulse durations (0.01 and 0.03 seconds), and application times (3 and 5 seconds), with irradiation conducted from a 4 mm distance from the bracket surface. Shear tests were performed using a universal testing machine at a crosshead speed of 0.5 mm/minute, and ARI was assessed. The results demonstrated that a laser setting of 10 W output, 0.01-second pulse duration, and three seconds application time was the most enamel-safe protocol, significantly reducing the debonding force compared to other tested settings.

Nd:YAG laser

Hayakawa conducted a study to enhance the debonding process of ceramic brackets using a high peak Nd:YAG laser at energy levels of 1.0, 2.0, or 3.0 J [36]. The experiment involved bonding both polycrystalline and monocrystalline ceramic brackets to extracted bovine mandibular incisor teeth with two different adhesives, Bis-GMA and MMA resin. The laser was applied as close as possible to the thinnest part of the bracket, specifically under each bracket wing and at the mesiodistal center of the gingival portion. Shear testing was performed, and the ARI was assessed. The findings indicated that there was no significant difference in the performance of the two adhesives. Notably, a significant reduction in SBS was observed only at laser energy levels of 2.0 and 3.0 J, with debonding occurring due to photoablation or thermal ablation, which caused a gas pressure explosion of the brackets. The study also cautioned about the potential increase in heat production, which could adversely affect the pulp tissues.

Han et al. explored the effectiveness of the Nd:YAG laser in debonding polycrystalline ceramic brackets [15]. Using 30 extracted premolar teeth, the brackets were bonded and then subjected to a Nd:YAG laser irradiation of 3 W for three seconds at a distance of 1 mm from the bracket center. The results demonstrated a significant decrease in SBS, with the laser debonding causing less damage to the enamel and preserving the enamel microstructure more effectively than traditional methods. The study concluded that the Nd:YAG laser is an efficient tool for the debonding of ceramic brackets.

In the same vein, Lai et al. assessed the performance of a pulsed mode Nd:YAG laser in debonding metal brackets using 50 extracted premolar teeth [37]. In this experimental setup, laser irradiation was accompanied by a force of 4.9 N until the brackets were detached. The findings showed that the Nd:YAG laser performed better with less enamel damage compared to conventional debonding methods.

In contrast, Feldon et al. investigated the effectiveness of the Nd:YAG laser in debonding metal brackets across 48 extracted premolar teeth [16]. After bonding the brackets and subjecting them to thermocycling in water for 1000 cycles, the brackets were irradiated with a Nd:YAG laser at outputs of 1.0, 1.5, or 2 W. The results did not show significant differences between the irradiated and non-irradiated groups. Despite the laser settings, all specimens irradiated with the laser exhibited an increased incidence of enamel cracks, and in some cases, irreversible adverse effects on the enamel were noted, challenging the safety of using Nd:YAG lasers for debonding metal brackets.

Tm:YAP laser

Dostalova et al. conducted a comparative study to evaluate the effectiveness of various laser types in debonding ceramic brackets, specifically examining a diode-pumped Tm:YAP laser (3.8 W, 1.997 µm), a diode gallium arsenide (GaAs) laser (20 W, 0.808 µm), and an Nd:YAG laser (2 W, 1.444 µm) [38]. The study involved 10 extracted premolar teeth to which polycrystalline ceramic brackets were bonded using a conventional adhesive system, followed by shear testing with a universal testing machine. Laser irradiation was applied for durations of 60 or 90 seconds, with and without water cooling. The findings demonstrated that the diode-pumped Tm:YAP and Nd:YAG lasers effectively debonded the brackets within 60 seconds, causing minimal enamel damage. The debonding process was primarily facilitated by thermal softening. However, the diode GaAs laser proved to be ineffective for this application.

Further insights into the debonding process were provided by another study from Dostalova et al., which focused on precoated ceramic brackets [39]. This research involved 20 extracted teeth (premolars and molars) where 10 used precoated brackets and 10 used self-etching adhesives. The brackets were subjected to diode-pumped Tm:YAP laser irradiation (3.8 W, 1.997 µm), followed by assessments of SBS and ARI. The results confirmed that thermal debonding was the primary mechanism involved. In a subsequent study by the same research group [19], their aim was to measure tensile strength, linking the debonding of ceramic brackets to thermal ablation following irradiation with the Tm:YAP laser.

In yet another study by Dostalova et al., the efficiency of the Tm:YAP laser under water cooling was tested using different protocols: 1 W for 60 seconds and 2 W for 60 seconds, on both precoated and polycrystalline ceramic brackets [40]. The optimal laser output was determined to be 1 W, which preserved the enamel microstructure intact across all tested groups. Consequently, the authors recommended the use of the Tm:YAP laser with water cooling for debonding ceramic brackets due to its efficacy and safety.

Thulium-doped (Tm) fiber laser

Demirkan et al. conducted a study to develop a debonding method for ceramic brackets that minimizes enamel cracks and patient discomfort [41]. For the first time, a Tm fiber laser with a wavelength of 1940 nm was used for this purpose, employing varying energy levels, outputs, and lasing durations. Polycrystalline ceramic brackets were attached to the labial surfaces of extracted mandibular incisor teeth using a conventional adhesive system. In the experimental group, the laser application involved different settings: (30 J energy, 3.0 W output, 10 seconds lasing), (25 J energy, 2.5 W output, 10 seconds lasing), and (21 J energy, 3.0 W output, 7 seconds lasing). The laser was applied either using a scanning method in a Z-shaped motion from the upper distal surface of the bracket wing to the opposite corner or a non-scanning method targeting directly at the center of the bracket (the thinnest portion). Following laser application, the brackets were subjected to shear testing, and the ARI was assessed. The findings indicated that the most significant reduction in SBS occurred with laser energy of 30 J. Adhesive breakdown was primarily due to thermal softening, and importantly, no carbonization of the adhesive was observed

Ytterbium fiber laser

Sarp and Gulsoy tested the efficacy of a ytterbium fiber laser in the debonding of ceramic brackets [42]. Polycrystalline ceramic brackets were affixed to the labial surfaces of bovine mandibular incisor teeth using Bis-GMA, following the manufacturer's instructions. In the experimental setup, a ytterbium fiber laser with a wavelength of 1070 nm was targeted at the center's thinnest part of the bracket. The laser was applied at different power levels, in both continuous and intermittent wave modes, ensuring an identical spot size by positioning the laser tip 15 cm away at a slight angle. The brackets were then subjected to shear tests using a universal testing machine, and the ARI was evaluated. The results demonstrated that the ytterbium fiber laser, noted for its compact size, offers promising capabilities for debonding ceramic brackets. Statistically significant reductions in SBS were observed in the groups treated with the laser, with the modulated mode yielding better outcomes than the continuous mode. The primary debonding mechanism was identified as the thermal softening of the adhesive.

Er,Cr:YSGG laser

Al-maajoun et al. explored the debonding efficacy of Er,Cr:YSGG and CO_2_ lasers on ceramic cylinders affixed to human dentin [20]. In their setup, 60 human teeth were prepared by exposing, etching, rinsing, and drying the dentin, onto which lithium disilicate cylinders were cemented using resin cement and stored in distilled water at 37°C for 24 hours. Laser settings included a 1 mm tip diameter with outputs of 4 W and 3 W for the Er,Cr:YSGG and CO_2_ lasers, respectively, targeting the ceramic surfaces for specific durations. The study found that shorter irradiation times with the CO_2_ laser correlated with lower forces required for debonding, though it also noted carbonization of the adhesive, evident as brown discoloration.

In another experimental study, Sedky and Gutknecht assessed the Er,Cr:YSGG laser for debonding metal brackets from thirty premolar teeth [21]. The teeth were prepared with conventional adhesives and subjected to either traditional plier debonding or laser application. The laser, operating with a 300 µm tip at 6 W and specific pulse settings, targeted the bracket-enamel interface from various angles until debonding occurred. This method demonstrated a longer debonding time compared to pliers but resulted in clean removal without residual adhesive, attributed to thermal ablation.

Mirhashemi et al. conducted a similar study on debonding ceramic brackets bonded to standardized composite blocks using Er,Cr:YSGG and Er:YAG lasers [22]. The blocks underwent preparation, bonding, and storage processes before being exposed to laser irradiation and thermocycling. Despite using sophisticated laser parameters, no significant differences in SBS or ARI were observed between the laser-treated and control groups, although the non-lased group exhibited more damage to the composite during debonding. These findings highlight the nuanced outcomes of laser debonding depending on the material and settings employed

Diode laser

**Figure 2:**
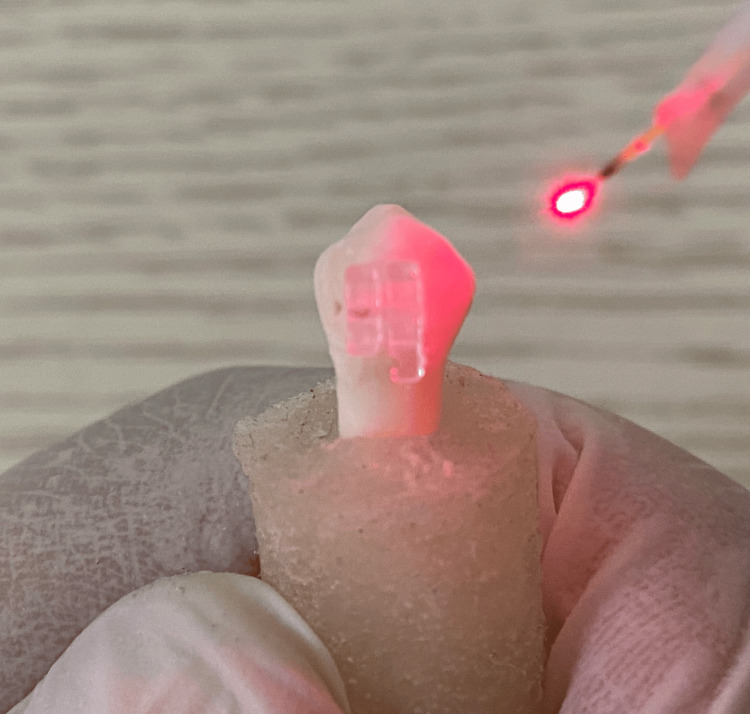
Close-up view of diode laser application for debonding ceramic bracket Image credit: Ahmed S. Khalil

In the majority of studies, the diode laser tip is typically positioned in close proximity to the brackets (Figure 2).

Feldon et al. explored the effectiveness of a diode laser in facilitating the debonding of ceramic brackets [16]. The study involved 60 bovine incisor teeth to which monocrystalline and polycrystalline ceramic brackets were bonded using a self-etching adhesive system as per the manufacturer's instructions. The diode laser, set at 2 W and 5 W, was applied directly above the bracket's metal slot for three seconds. Shear tests and ARI measurements followed. Results indicated a significant reduction in SBS for the lased monocrystalline brackets compared to non-lased groups, with no notable difference between the two laser settings. Conversely, polycrystalline brackets showed no significant changes in SBS between lased and non-lased groups due to their less uniform crystal structure and reduced transparency. ARI results remained consistent across groups.

In a contrasting study, Ivanov assessed the impact of diode laser debonding on ceramic brackets [28]. Both monocrystalline and polycrystalline ceramic brackets were bonded to bovine incisor teeth using a self-etching adhesive. An 820 nm diode laser was applied at the bracket-enamel interface for three and six seconds at 2.5 and 5 W, respectively. Shear testing and ARI evaluation followed. The findings indicated no advantage of laser use for monocrystalline brackets, whereas polycrystalline brackets showed significant reductions in SBS and increases in ARI scores, particularly with the 2.5 W setting over six seconds.

Almohaimeed and Abdelhalim conducted a study to determine an efficient protocol for debonding precoated ceramic brackets [17]. The use of precoated brackets was aimed at standardizing adhesive thickness and reducing technique sensitivity and errors. Eighty human premolar teeth were prepared and bonded with two types of precoated ceramic brackets, APC II and APC Plus (3M Company, Saint Paul, Minnesota, United States), and stored for 48 hours. Laser application was performed for three seconds using a 980 nm wavelength and 3 W output. After 24 hours, shear testing and ARI assessments were conducted. The results showed a significant decrease in SBS and an increase in ARI scores in the laser-treated groups, suggesting a lower risk of enamel damage.

Yassaei et al. conducted a study to evaluate the characteristics of enamel surface post-debonding of ceramic brackets [43]. Thirty extracted human premolars were prepared and bonded with polycrystalline ceramic brackets using a conventional adhesive system, followed by storage in water for 24 hours. Debonding was carried out using pliers, with one group pre-treated with diode laser irradiation. The laser was applied in a continuous mode, sweeping parallel to the bracket slot from a 5 mm distance for a total of 10 seconds. The laser settings used were 2.5 W and 980 nm wavelength. The laser-treated group showed statistically fewer and shorter enamel cracks compared to the control group, although there was no significant difference in ARI scores between the groups.

In a related study by Yassaei et al., the impact of various diode laser settings on enamel surface integrity was tested using 90 extracted human premolars [19]. Brackets were bonded using the conventional method and subjected to different laser outputs (2, 2.5, and 3 W) and modes (continuous and pulsed) for 10 seconds. The wavelength used was 980 nm with a pulse duration of 30 microsecond (µs). Despite the various settings, all laser treatments significantly increased the frequency of enamel cracks, with no substantial difference between the pulsed and continuous modes. Approximately 65% of the samples achieved ARI scores of 2 or 3, indicating varied adhesive remnants on the enamel.

Stein et al. explored the debonding efficacy of ceramic brackets using a 445 nm diode laser [29]. The study included 15 extracted human third molars, comparing two aspects, treated with and without laser. Prior to irradiation, brackets were marked with a black crayon to enhance laser absorption. The laser was applied perpendicularly to the bracket bases for 15 seconds in total, and laser parameters were as follows: tip diameter: 320 µm, output: 2 W, and continuous mode. Post-debonding ARI assessments and enamel examinations showed no fractures in the laser group, unlike the control group which experienced some bracket fractures. The laser group also displayed significantly lower ARI scores.

Anand et al. investigated the time lag and its impact on SBS and enamel integrity [18]. Sixty extracted premolars were prepared and bonded with precoated polycrystalline ceramic brackets. The laser was applied for five seconds with settings of 2.5 W and 810 nm wavelength. Debonding was performed either immediately or one hour after lasing. The results indicated a significant reduction in SBS for laser-treated groups, attributed to thermal softening. The immediate and delayed debonding showed no significant difference. Yet, immediate debonding resulted in higher ARI scores. An increased presence of enamel cracks was observed in the non-lased group under scanning electron microscopic examination.

Sinaee et al. assessed the impact of diode laser debonding on enamel properties [44]. Twelve extracted premolars were prepared, and monocrystalline ceramic brackets were bonded on multiple surfaces. After water storage and thermocycling, a 940 nm diode laser was applied at 1 W or 3 W for three seconds. Post-debonding tests included shear testing, ARI recording, and nanoindentation to measure enamel hardness and elasticity. The study found a significant decrease in SBS in laser-treated groups, with no notable difference between 1 W and 3 W settings. However, there were no significant differences in ARI scores or the enamel properties.

Er:YAG laser

Tocchio et al. explored the debonding effects of Er:YAG laser on both monocrystalline and polycrystalline ceramic brackets [25]. The study involved bovine deciduous incisors which were prepared and bonded using a conventional adhesive system. Laser application varied in power density, with outputs ranging from 3-33 W across wavelengths of 248, 308, and 1060 nm. A debonding force of 0-0.8 megapascal (Mpa) was applied using stainless steel ligature wire. The study observed that while polycrystalline brackets required up to 24 seconds to debond at different wavelengths, most monocrystalline brackets debonded in under a second. Both types of brackets were debonded without any enamel damage, with thermal softening and adhesive decomposition identified as the mechanisms for polycrystalline brackets, whereas thermal ablation was determined to be responsible for the debonding of monocrystalline brackets.

Following a similar investigation, Suh et al. assessed the efficacy of Er:YAG laser for debonding ceramic brackets across 190 teeth [26]. Laser irradiation varied from 0 to 600 mJ, applied at two points on each bracket. Their findings indicated a significant decrease in SBS as laser energy increased, identifying 450 mJ for monocrystalline and 300-450 mJ for polycrystalline brackets as the optimal energies for efficient and safe debonding.

Oztoprak et al. introduced a laser scanning method to evaluate the Er:YAG laser’s impact on debonding polycrystalline ceramic brackets [24]. Sixty bovine mandibular incisors were prepared and bonded accordingly. Using a 4.2 W laser at a 2940 nm wavelength, irradiation was conducted for nine seconds in a scanning pattern, followed by shear testing. This method resulted in a significant reduction in SBS and ARI scores, suggesting less enamel damage and highlighting the advantages of using a scanning method with Er:YAG for debonding.

Nalbantgil et al. [23] explored the effect of varying laser irradiation durations on the debonding process. Eighty bovine incisors were prepared and bonded with polycrystalline ceramic brackets, then subjected to 3, 6, or 9 seconds of Er:YAG laser irradiation using a scanning approach. Their results favored a 6-second irradiation duration for optimal debonding with minimal enamel damage.

A follow-up study by Nalbantgil et al. compared the outcomes of using Er:YAG laser with and without water cooling [45]. Sixty human premolars were prepared and bonded, and laser irradiation was performed for nine seconds. Both conditions showed acceptable SBS values, but the use of water cooling was recommended to reduce the risk of intrapulpal injury.

Tozlu et al. studied the impact of time lag between lasing and debonding on the efficiency of ceramic bracket removal [46]. One hundred human premolars were prepared and bonded, and subjected to Er:YAG laser irradiation from 2 mm distance followed by debonding at various time lags of one, 18, 30, or 60 seconds. The 18-second lag provided the most reliable clinical results, with improved ARI scores suggesting less risk of enamel damage.

Mundethu et al. evaluated the potential of a single pulse Er:YAG laser irradiation for rapid debonding of ceramic brackets [47]. This innovative approach was tested on 20 human permanent molar teeth, where the laser was positioned as close as possible to the bracket slot. Remarkably, 19 out of 20 brackets were rapidly removed with an audible sound, illustrating the effective use of photoablation and thermomechanical ablation induced by the laser. This method also highlighted the need for caution in clinical settings to prevent accidental ingestion of debonded brackets.

Dostalova et al. investigated the debonding efficacy of the Er:YAG laser on both metal and ceramic brackets [48]. In their study, 30 extracted teeth were divided into three groups based on the type of brackets and adhesives used: metal brackets and polycrystalline ceramic brackets bonded with self-etching adhesive, along with polycrystalline ceramic brackets bonded using a conventional adhesive system. Laser irradiation was performed under long pulse mode for 140 seconds at settings of 4 mm tip diameter, 2940 nm wavelength, 280 mJ energy density, 6 hertz (Hz) frequency, and 250 µs pulse duration, employing both air and water cooling. Post irradiation, brackets were removed with a band-removing plier, and the enamel surface was assessed under an electron microscope. The outcomes demonstrated a notably easier removal of brackets in the laser groups without enamel damage, supporting the thermal softening hypothesis as the mechanism facilitating debonding

In a similar vein, Sabuncuoglu et al. assessed the effectiveness of the Er:YAG laser in debonding polycrystalline ceramic brackets [49]. They prepared 20 extracted lower incisors, storing them in distilled water at 37 ºC for 48 hours prior to treatment. The laser parameters for the irradiation involved a 3 W output, 2940 nm wavelength, 120 mJ energy density, 10 Hz frequency, and 100 µs pulse duration, with the addition of air and water cooling. The brackets were tested for shear strength immediately after laser application, and the ARI was measured. Results indicated a significant reduction in SBS and an increase in ARI scores, suggesting less enamel damage due to thermal softening.

Furthering this research, Yilanci et al. tested the efficiency of the Er:YAG laser in debonding ceramic brackets, examining the process under both standard and thermocycled conditions [50]. Forty extracted human teeth, comprising 20 maxillary central incisors and 20 premolars, were etched, rinsed, and dried. Monocrystalline ceramic brackets were bonded and stored in distilled water for 24 hours. The laser debonding was executed using a 1.3 mm tip diameter, 1.2 W output, 2940 nm wavelength, 600 mJ energy density, 2 Hz frequency, and 250 µs pulse duration. After the initial debonding, adhesive remnants were cleared and the teeth were polished. The same teeth were then subjected to thermocycling between 5 ºC and 55 ºC for 5000 cycles. The study revealed a slight decrease in SBS due to adhesive hydrolysis in the thermocycled group. Yet, no significant differences were found regarding pulp safety, affirming the laser's safety and efficacy in the debonding process.

Discussion

The development of ceramic brackets was driven by the increasing demand among adult patients for aesthetically appealing orthodontic solutions. However, debonding ceramic brackets often involves excessive force and patient discomfort. Given the limited research on optimal debonding techniques, this review aims to explore various lasers used for this purpose and to summarize the relevant literature. The primary finding of this review, highlighted by multiple studies, is that Er:YAG and Er,Cr:YSGG lasers exhibit lower thermal effects compared to other lasers and are readily absorbed by tissues containing water [27]. Consequently, orthodontic adhesives effectively absorb the energy emitted by these lasers which makes them the lasers of choice to facilitate debonding ceramic brackets [51].

Studies such as these by Mundethu et al. [47] and Sedky and Gutknecht [21] found that continuous lasing with Er:YAG and Er,Cr:YSGG, respectively, without external force can cause ceramic brackets to detach spontaneously. This debonding effect is attributed to the photoablation mechanism, where the uncured monomer and water in the adhesive absorb the laser energy, expand, and vaporize [52]. This process generates subsurface pressure leading to microexplosions that reduce the SBS of the brackets in the micro-level. This is opposed to the thermal softening that was proposed with the use of diode lasers [16,17]. The resultant high ARI indicates minimal enamel damage, albeit with the downside of increased chair time for adhesive removal [53,54]. Also, the large size and the increased lasing time of Er:YAG and Er,Cr:YSGG lasers remain significant disadvantages. Furthermore, Er:YAG lasers have shown efficacy in the prosthodontic field, significantly reducing the SBS of laminate veneers and facilitating their removal with minimal impact on the enamel [55-60].

Despite the compact size of diode lasers making them a staple in daily practice, their effectiveness remains debated due to the lack of standardized protocols and clear guidelines. Variations in study designs, including differences in power, wavelength, lasing duration, sample size, and adhesive types, contribute to this uncertainty [16,28].

Overall, debonding forces of 6-8 MPa are necessary to remove orthodontic brackets effectively [61]. Forces beyond this range can cause enamel tear-outs and irreversible damage, whereas insufficient forces may lead to premature bracket failure [62]. That being said, high ARI scores consistently indicate reduced enamel damage, yet the necessity for extended chair time for adhesive removal is an ongoing challenge, particularly detected with the use of Er:YAG and Er,Cr:YSGG lasers.

Limitations

The limitations of this review need to be recognized. Caution is advised in interpreting the results given the substantial methodological diversity among the studies, including variations in laser output, wavelength, mode, lasing time, time lag between lasing and debonding, and type of adhesive used. Additionally, this review only considered studies published in English, possibly omitting pertinent research published in other languages.

Recommendations for Future Research

Further research is needed to establish a standardized protocol for laser-aided debonding of ceramic brackets. Additionally, clinical trials are crucial to compare different techniques of debonding and confirm the efficacy of these methods prior to their routine application. Further well-conducted high-quality research is needed to assess the safety of lasers on dentition and pulp tissues.

## Conclusions

Er:YAG and Er,Cr:YSGG could be the lasers of choice for debonding ceramic brackets due to their demonstrated efficiency. However, their large size presents a significant drawback, limiting their practicality in some clinical settings. Rigorous comparison between lasers used for debonding ceramic brackets is challenging due to the high heterogeneity, relatively low-level evidence, and diverse outcome variables across the studies. 
